# In vitro performances of a valved holding chamber with inhaled corticosteroids

**DOI:** 10.1186/1939-4551-8-S1-A195

**Published:** 2015-04-08

**Authors:** Nabile Boukhettala, Thierry Porée, Lissbeth Leon-Bollotte

**Affiliations:** 1 Laboratoire Protec'som, France

## Background

In young children with asthma, it is recommended to use pressurised metered dose inhaler (PMDI) with a valved holding chamber (VHC). The objective of this study was to evaluate the performances of a VHC with inhaled corticosteroids.

## Methods

In this study, the VHC called Tipshaler (Protec’som, France) was evaluated with fluticasone (Flixotide®, 50µg/dose, GSK, France) and beclomethasone (QVAR®, 100µg/dose, MEDICIS, Canada). The method according to the European Pharmacopoeia used a constant flow rate (30 L / min) was used. Particle size distribution was measured using a NGI cascade impactor (Copley Scientific, Nottingham, United Kingdom). The fluticasone and beclomethasone concentrations were assayed by spectrophotometry at 236 nm and 239 nm respectively.

## Results

In the trachea, the mass of fluticasone was higher with pMDI alone in comparison with VHC (20 ± 0,6 μg vs 0,9 ± 0,3 μg, p <0,05). The fine particle dose of fluticasone was similar with pMDI alone compared to VHC (26 ± 2 μg vs 24 ± 1 μg). Concerning beclomethasone, in the trachea the mass of drugs was higher with pMDI alone in comparison with VHC (11,6 ± 0,4 vs 1,2 ± 0,2, p<0.05). In addition, deposition of fine particles of beclomethasone was similar with pMDI alone in comparison with VHC (77 ± 1 µg vs 75 ± 1 µg, p<0.05).

## Conclusions

The use of valved holding chamber reduces the deposition of particles of inhaled corticosteroids in the trachea and allows efficient lung deposition of drugs.

